# Artemisinin reduces PTSD-like symptoms, improves synaptic plasticity, and inhibits apoptosis in rats subjected to single prolonged stress

**DOI:** 10.3389/fphar.2024.1303123

**Published:** 2024-02-06

**Authors:** Qing Liu, Xiaoyan Ding, Ying Wang, Hairong Chu, Yan Guan, Meng Li, Kuisheng Sun

**Affiliations:** School of Laboratory Medicine, Weifang Medical University, Weifang, Shandong, China

**Keywords:** post-traumatic stress disorder, artemisinin, single prolonged stress, synaptic plasticity, apoptosis

## Abstract

Post-Traumatic Stress Disorder (PTSD) is a chronic mental disorder characterized by symptoms of panic and anxiety, depression, impaired cognitive functioning, and difficulty in social interactions. While the effect of the traditional Chinese medicine artemisinin (AR) on PTSD is unknown, its therapeutic benefits have been demonstrated by studies on models of multiple neurological disorders. This study aimed to extend such findings by investigating the effects of AR administration on a rat model of PTSD induced by a regimen of single prolonged stress (SPS). After rats were subjected to the SPS protocol, AR was administered and its impact on PTSD-like behaviors was evaluated. In the present study, rats were subjected to a multitude of behavioral tests to evaluate behaviors related to anxiety, memory function, and social interactions. The expression of hippocampal synaptic plasticity-related proteins was detected using Western blot and immunofluorescence. The ultrastructure of synapses was observed under transmission electron microscopy. The apoptosis of hippocampal neurons was examined with Western blot, TUNEL staining, and HE staining. The results showed that AR administration alleviated the PTSD-like phenotypes in SPS rats, including behavior indicative of anxiety, cognitive deficits, and diminished sociability. AR administration was further observed to improve synaptic plasticity and inhibit neuronal apoptosis in SPS rats. These findings suggest that administering AR after the onset of severe traumatic events may alleviate anxiety, cognitive deficits, and impaired social interaction, improve synaptic plasticity, and diminish neuronal apoptosis. Hence, the present study provides evidence for AR’s potential as a multi-target agent in the treatment of PTSD.

## 1 Introduction

Induced by traumatic experiences, post-traumatic stress disorder (PTSD) is a devastating psychiatric condition characterized by heightened arousal and the continual recollection of stressful memories linked to the trauma (Compean and Hamner, 2019). While approximately 6%–7% of the population will experience PTSD at some point in their lives, the prevalence of PTSD is notably higher among individuals who have undergone particularly traumatic events, such as war and violent crime (Schrader and Ross, 2021). PTSD is associated with anxiety and deficits in social interaction, cognition, and memory (Wilkins et al., 2021). Among them, anxiety is a common symptom in patients with PTSD (Sadeghi et al., 2024). PTSD-related anxiety symptoms stem from the generalization of individual’s initial fear memory, which can trigger anxiety reactions even when exposed to backgrounds similar to the original trauma background (Dunsmoor and Paz, 2015). PTSD is also often associated with significant cognitive impairment. Current PTSD theory suggests that cognitive abnormalities are the core of the occurrence and persistence of PTSD symptoms (Pitts et al., 2022). Social deficits are another typical symptom of PTSD. Patients avoid social situations and have difficulty maintaining positive interpersonal relationships (Mittal et al., 2013; Gou et al., 2023). Preclinical studies have identified synaptic plasticity changes and increased rates of apoptosis as significant pathophysiological mechanisms underlying PTSD (Gu et al., 2023; Xie et al., 2024). While various treatment options, such as psychotherapy and pharmacotherapy, are available for patients with PTSD, much work remains to improve the effectiveness and accessibility of these treatments, as well as clarify the causes that underlie the neuropathological consequences of trauma (Quinones et al., 2020; Astill et al., 2021).

The relationship between the dysfunction of the prefrontal cortex-hippocampus-amygdala circuit related to learning and memory, and the pathological changes of PTSD has long been a focus of attention (Alexandra et al., 2022). The amygdala is the emotional control center of the brain, especially emotions related to fear and anxiety (Simic et al., 2021). The prefrontal cortex is involved in emotional regulation and cognitive control in PTSD (Alexandra et al., 2022). The hippocampus is an important brain region in this circuit, responsible for the key structures of memory storage and retrieval, especially memories related to emotions and trauma (Bremner, 2006; Fenster et al., 2018; Logue et al., 2018; Wang et al., 2022). In PTSD patients, structural and functional changes occur in the hippocampal region, leading to abnormal storage and recall of traumatic memories. This, in turn, results in individuals repeatedly recalling the traumatic event and being unable to shake off negative emotions (Shin et al., 2006; Tural et al., 2018; Bremner et al., 2021). Therefore, studying the role of the hippocampal region in PTSD is conducive to understanding the mechanism of the disease and developing potential therapeutic strategies.

Artemisinin (AR) is a natural compound obtained from the Artemisia annua plant. Although AR is most renowned for its potent anti-malarial properties (Ma et al., 2020), recent findings suggest that it exerts neuroprotective effects (Kshirsagar and Rao, 2021; Arthur et al., 2023). Specifically, it may alleviate oxidative stress, inhibit neuroapoptosis, suppress neuroinflammation, and promote synaptic plasticity in the contexts of multiple neurological conditions, including stroke, Alzheimer’s disease, Parkinson’s disease, and depression (He et al., 2021; Yan et al., 2021; Peng et al., 2022). However, the potential of AR to act as a neuroprotective agent in patients with PTSD, particularly to improve synaptic plasticity and neuronal apoptosis in affected individuals, remains largely unexplored. To resolve this dearth in the literature and inform future directions in the treatment of PTSD, our study explores the neuroprotective effects of AR on PTSD-like phenotypes in rats exposed to prolonged stress.

The single prolonged stress (SPS) procedure is a widely accepted preclinical paradigm commonly used to simulate PTSD-like behavior in rodents. By subjecting them to a combination of severe and inescapable stressors such as immobilization, forced swimming, and ether exposure, it aims to cause sufficient trauma to the animals to induce enhanced and long-lasting PTSD-like symptoms (Souza et al., 2017; Lisieski et al., 2018; Richter-Levin et al., 2019). Our central hypothesis posits that AR can induce the enhancement of synaptic plasticity and inhibit neuronal apoptosis, thereby attenuating the symptoms of PTSD. The current study used the SPS procedure to simulate PTSD in rodents. A series of tests were then conducted to assess the impact of AR on the rodents’ behaviors: the Morris water maze (MWM) test to investigate spatial exploration and memory function, the elevated plus-maze (EPM) to evaluate anxiety-like behaviors, the open field test (OFT) to gauge exploratory behaviors, and the three-chamber social interaction test (SIT) to assess social communication. The present study then measured the effect of AR on the synaptic morphology, synaptic-associated proteins, and neuronal apoptosis in the hippocampal region of SPS-induced rats. The findings obtained from this comprehensive investigation may offer insight into the potential therapeutic impact of AR on PTSD, as well as novel, effective treatments for the disorder.

## 2 Materials and methods

### 2.1 Animals

Due to the neuroprotective effects of estrogen, only male SD rats were used in this study (Wang et al., 2019). Specific pathogen-free male SD rats (weighing 200–250 g) were habituated to a 12-h light/dark cycle and allowed to eat and drink *ad libitum*. Room temperature was maintained at 20°C–25°C. All behavioral tests were conducted between 8:00 a.m. and 11:00 a.m. The experimental protocol was approved by the Animal Ethics Committee of Weifang Medical University on 1 January 2021 (approval number: 2021SDL577).

### 2.2 Experimental animal grouping

**FIGURE 1:**
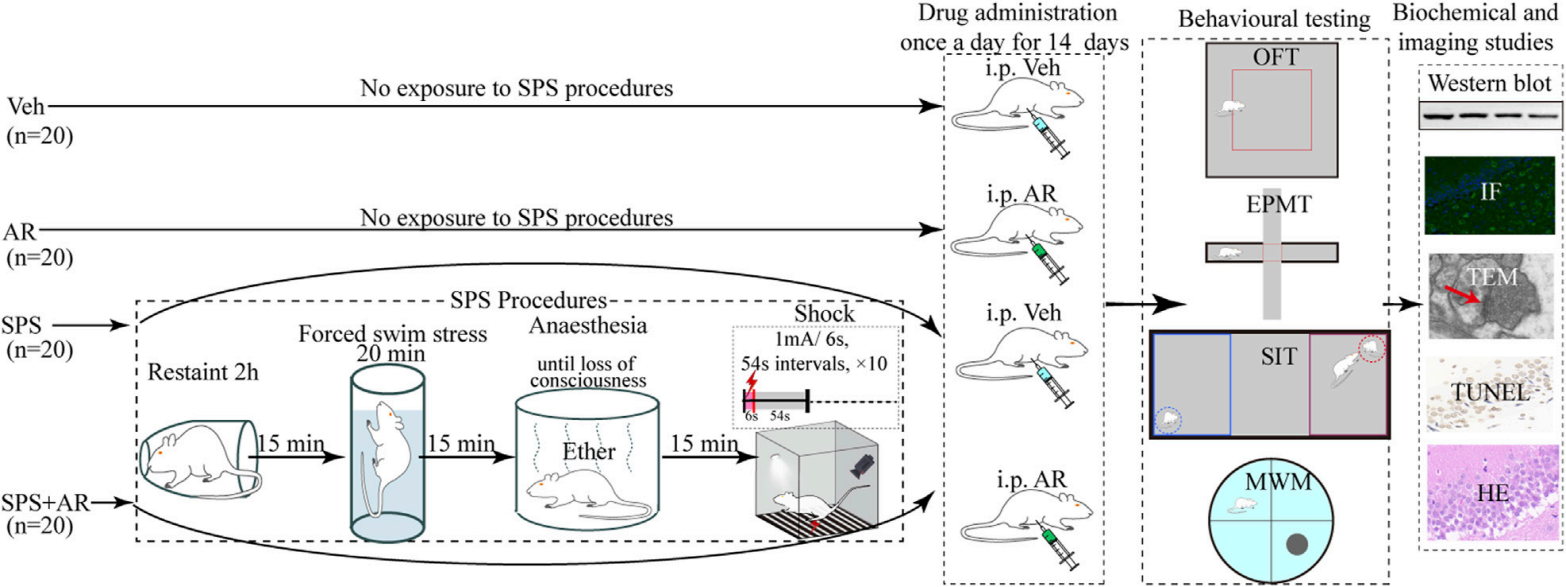
In this study, we constructed a schematic of the PTSD model through the SPS procedure in rats, AR or saline administration modality and the four behavioral paradigms of MWM, EPM, OFT, and SIT. Specifically, following the completion of the aforementioned four behavioral tests by all animals, biochemical and imaging studies were conducted. For these studies, five animals were randomly selected from each group for Western blot analysis, five for imaging studies, and five for TEM studies.

In this study, artemisinin was administered to rats at a concentration of 18 mg/kg via intraperitoneal injection (i.p.), as described in previous literature (Lv et al., 2023). As shown in Figure 1, eighty rats were randomly divided into four groups of 20: the SPS + AR group underwent SPS followed by AR administration once a day for 14 consecutive days, the vehicle (Veh) group did not undergo SPS and received saline once a day for 14 consecutive days, the AR group did not undergo SPS and received AR once a day for 14 consecutive days, and the SPS group underwent SPS stimulation followed by saline administration once a day for 14 consecutive days. The animals were housed separately according to their respective groups. The AR used in this study was obtained from Sigma-Aldrich (St. Louis, MO, United States of America; CAS 63968-64-9; molecular weight, 282.33 g/mol; purity, ≥98.0%).

### 2.3 SPS procedure

This study used a typical SPS rat model to simulate trauma experienced by humans. As previously reported (Torok et al., 2019), SPS begins with restraint stress, where the animal is restrained for 2 h to simulate helplessness and immobilization. Fifteen minutes afterward, the animal is placed in a tank filled with water and forced to swim for 20 min to stay afloat (water temperature: 25°C ± 2°C). Fifteen minutes later, the animal is exposed to ether vapor for 15 min to induce anesthesia. Thirty minutes afterward, the animal is exposed to a tone followed by a mild electric foot shock (1 mA for 6 s, 10 times) to induce a fear response (Figure 1).

### 2.4 Behavioral tests

As shown in Figure 1, all 80 rats in this study were subjected to OFT, EPMT, three-chamber SIT, or MWM test 1 day after administration of AR or saline to monitor the PTSD-associated behavioral and physiological changes, including anxiety, social interaction skills, and spatial learning memory abilities. Throughout the testing, the investigators remained at a minimum distance of 1 m from the equipment while they recorded data for each group. Once each test was completed, the investigator returned the rats to their feeding cages and sanitized the equipment with 75% alcohol to prevent the transmission of any residual information (e.g., urine, feces, and odor) to subsequently tested rats.

### 2.5 OFT

The OFT was performed in a large, brightly lit open arena (100 × 100 × 50 cm) whose walls were devoid of any visual or tactile cues. The procedure consisted of the following steps. The camera was positioned directly above the arena to capture the rats’ paths of movement. The rats were allowed 10 min of free exploration in the center of the arena. The behaviors of the rats were then observed and recorded for 5 min. Data analysis was performed with Smart 3.0 (Barcelona, Spain).

### 2.6 EPMT

The elevated plus-maze was elevated to a height of 70 cm above the ground. The apparatus contained four closed arms of 50 cm in length and 10 cm in width. Two of the arms were enclosed at a height of 40, and two were left uncovered. Before the experiment, each rat was allowed to acclimatize to the test chamber for ≥20 min. The experiment began with the rat placed at the center of the maze and facing the closed arm. Its subsequent movements were then recorded for 5 min, during which time the rat’s number of entries into and durations of stay in the open and closed arms were recorded. After the test, the rats were removed from the maze and returned to their respective cages. The open-arm entries and open-arm dwell time percentages were computed to evaluate each tested rat’s level of anxiety.

### 2.7 Three-chamber SIT

As described previously (Xiao et al., 2022), the experimental arena comprised a topless rectangular box (120 cm × 60 cm × 40 cm) divided into three chambers of equal volumes (left, middle, and right). The chambers were connected by two doors (20 cm × 10 cm) on either wall of the middle volume. The rats were allowed to freely pass between the three parts. After being placed in the empty middle chamber of the device, the tested rats were allowed to freely explore each chamber for 10 min. After this habituation period, a new rat (referred to as unfamiliar 1 [U1]) was placed in a confinement cage located in the right chamber (referred to as chamber U1), while the confinement cage in the left chamber (referred to as chamber E) was left empty. The tested rat was placed in the central chamber and was allowed to wander freely for 10 min (the sociability phase). Following that, a new and unfamiliar rat (unfamiliar 2 [U2]) was placed in the confinement cage in the left chamber (referred to as chamber U2). The U1 rat, which had previously been used in the sociability phase, was left in its original position as a familiar animal. Subsequently, the rats were permitted to explore the testing environment without any limitations for 10 min (the social novelty preference phase). Behavioral data such as rat movement trajectories, time spent in each of the chambers, and time spent interacting with unfamiliar rats or empty chambers were analyzed to assess sociability and social novelty preference during both phases of the test.

### 2.8 MWM test

The MWM experiment was conducted in a circular pool divided into four quadrants, one of which (the target quadrant) contained a hidden platform submerged to a depth of approximately 2 cm beneath the water. The escape latency—i.e., the time the animal takes to find the hidden platform within 1 min—was automatically recorded by the software of Smart 3.0 during the 5-day locomotor memory learning process. If the platform was not found within 1 min, the observer would direct the animal to climb onto it and remain there for 20 s; in this event, the escape latency was recorded as 60 s. During the 5-day training period, each rat received three training sessions per day. The spatial exploration test was conducted on the sixth day following the removal of the platform. Automated video tracking was used to record the length of time the rats spent in the target quadrant, the number of times they crossed the target quadrant, and the patterns of their movements.

### 2.9 Western blot

Rat hippocampal tissues were extracted from freshly collected rat brains and homogenized on ice in lysis buffer using the Total Protein Extraction Kit (KGP2100, KeyGEN Biotechnology Co., Ltd., Jiangsu, China), which contains phosphatase inhibitors, protease inhibitors, and phenylmethanesulphonyl fluoride. The homogenized tissues were then centrifuged at −4°C. After the total protein concentration was measured with a BCA assay kit, the protein extract was denatured with a loading buffer containing SDS and boiled. Samples were laid on 10% acrylamide gels and subjected to electrophoresis. Separated proteins were transferred onto polyvinylidene difluoride membranes, incubated with primary and secondary antibodies (Table 1), and visualized using ECL reagent. Membranes were imaged in a chemiluminescence apparatus and saved as TIFF images. The band optical density was then quantified and standardized using ImageJ software. The above Western blot method was based on previous literature with minor modifications (Gao et al., 2019).

**TABLE 1:** Antibody information about the method of Western blot.

Primary antibody	Place of origin	Item no.	Molecular weight (KDa)	Source of antibody	Dilution ratio of primary antibody	Secondary antibody	Place of origin	Item no.	Dilution ratio of primary antibody
GAPDH	Servicebio	GB15002	37	Mouse	1: 2000	HRP-goat anti-mouse	Servicebio	GB23301	1: 5000
β-tubulin	Servicebio	GB122667	55	Mouse	1: 2000	HRP-goat anti-mouse	Servicebio	GB23301	1: 5000
BDNF	ABCAM	ab205067	15	Mouse	1: 2000	HRP-goat anti-mouse	Servicebio	GB23301	1: 5000
Synapsin I	ABCAM	ab254349	75	Rabbit	1: 1000	HRP-goat anti-rabbit	Servicebio	GB23303	1: 5000
PSD95	ABCAM	ab18258	80	Rabbit	1: 1000	HRP-goat anti-rabbit	Servicebio	GB23303	1: 5000
β-actin	Servicebio	GB12001	42	Mouse	1: 2000	HRP-goat anti-mouse	Servicebio	GB23301	1: 5000
BAX	ABCAM	AB32503	20	Rabbit	1: 1000	HRP-goat anti-rabbit	Servicebio	GB23303	1: 5000
BCL2	ABCAM	AB196495	26	Rabbit	1: 1000	HRP-goat anti-rabbit	Servicebio	GB23303	1: 5000
CASPASE3	ABCAM	AB184787	35	Rabbit	1: 1000	HRP-goat anti-rabbit	Servicebio	GB23303	1: 5000

### 2.10 Experimental analysis of morphology

In this study, we performed three molecular imaging studies: immunofluorescence (IF), hematoxylin and eosin staining (HE), and terminal deoxynucleotidyl transferase dUTP nick end labeling (TUNEL) staining. Five rats were randomly chosen from each group and anesthetized with pentobarbital sodium. After perfusing the heart of each rat with saline, the brain was carefully extracted and fixed with a 4% paraformaldehyde solution. A precision cryostat was used to slice brain sections of 4 μm in thickness. Three consecutive sections were taken from the hippocampal region of the rat brain and subsequently used for IF, HE, and TUNEL staining, respectively. After the sections were made (the specific sectioning process will be described in detail below), images were captured with a Leica DM6 microscope (Leica, Germany) and analyzed for fluorescence intensity or the number of positive cells compared with ImageJ software according to the specific instructions listed in Ref(Jensen, 2013). All rat hippocampal sections were analyzed by a technician (ML), who performed a blinded analysis on the data for each experimental group.

Before IF staining was performed, sections were washed with PBS, permeabilized with 0.2% Triton X-100 solution, and incubated in 5% bovine serum albumin to block non-specific binding. They were then incubated with primary antibody against BDNF (Abcam, ab205067, 1:500) overnight at 4°C, followed by incubation with goat anti-mouse 488-conjugate secondary antibody (Abcam, ab150113, 1:500) at 37°C for 1 h. Cell nuclei were then subjected to DAPI (Solarbio, S2110) staining. The sample slides were covered with Antifade Mounting Medium before images of them were obtained using a fluorescence microscope (Mirchandani-Duque et al., 2022).

HE staining was performed by staining the sections with hematoxylin for 3 min and subsequently with eosin for 1 min before dehydration. Images of the hippocampal region were captured under the DM6 microscope (Yu et al., 2022).

TUNEL staining began by incubating the sections in TUNEL reaction mixture in the dark for 60 min at a temperature of 37°C. Apoptotic cells that stained positively for TUNEL appeared brown and were counted under the DM6 microscope (Yu et al., 2022).

### 2.11 Transmission electron microscopy (TEM)

After anesthetizing the rats with pentobarbital sodium, their brains were promptly excised and the hippocampus was sectioned into 1 mm blocks. These tissues were immobilized in a TEM-specific solution (Cat#G1102, Servicebio) for 2 hours and subsequently washed twice with PBS. To enhance contrast, the tissues were stained with osmium acid, dehydrated using an alcohol-acetone mixture, and then embedded in resin. Further staining with uranyl acetate and lead prepared the samples for examination under a TEM using a Hitachi device. Quantitative analysis of the synaptic parameters, including synaptic length of active zone, synaptic interface curvature, synaptic cleft width, and post-synaptic density, was performed using ImageJ software (Cui et al., 2023).

### 2.12 Statistical analysis

The data were analyzed with SPSS 22.0 and GraphPad Prism 8.0. The mean value ± the standard error (SE) was used to express the data. Statistical analysis of the data was performed using one-way analysis of variance (ANOVA), after confirming the normality of the relevant datasets. We then performed *post hoc* tests using least significant difference (LSD) for pairwise comparisons. The statistical analysis was deemed significant at a level of *p* < 0.05.

## 3 Results

### 3.1 Effects of AR administration on SPS-induced anxiety-like behaviors, social interaction behaviors, and learning and memory behaviors

**FIGURE 2:**
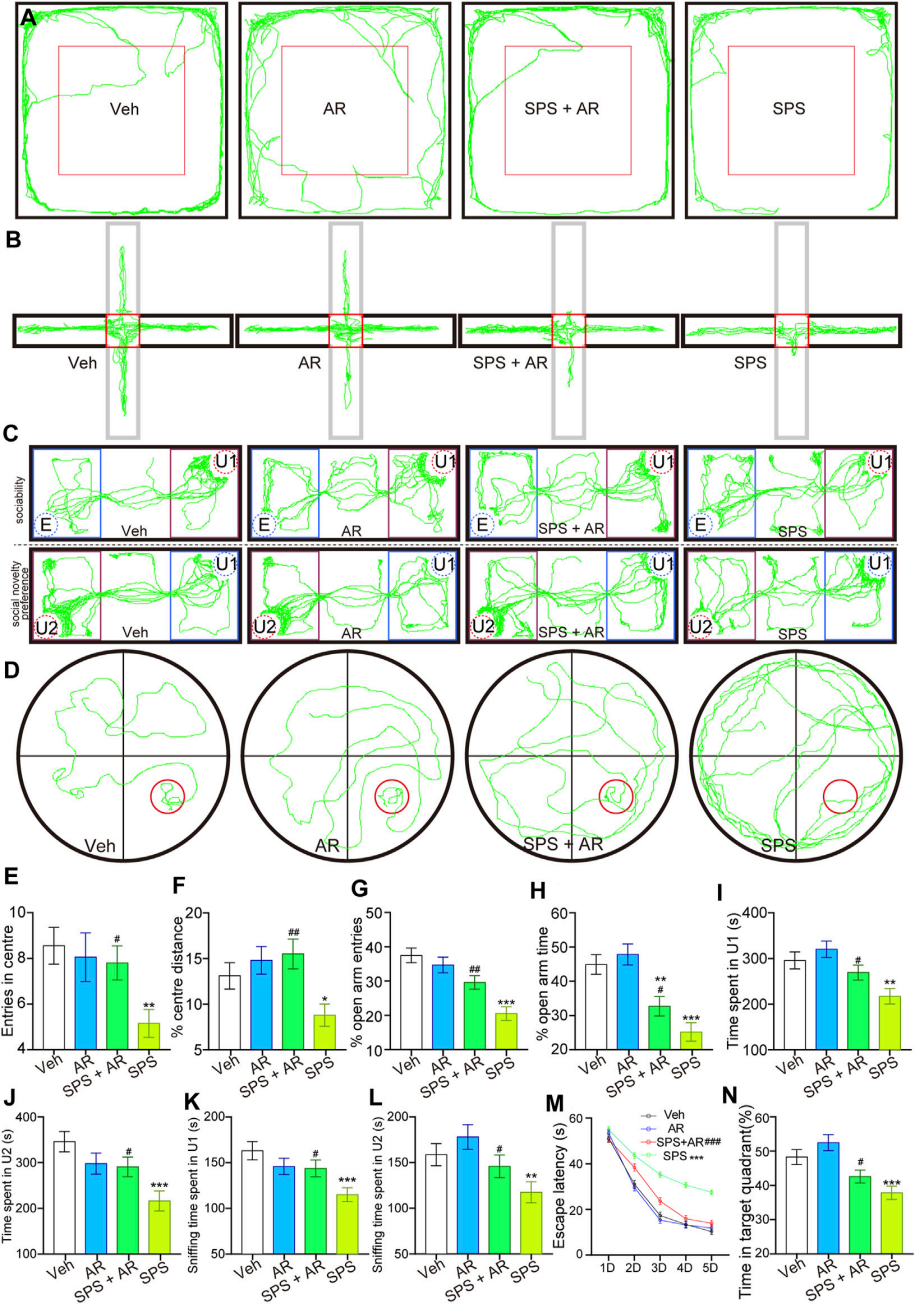
AR administration mitigated anxiety-like behaviors, social aversion, and learning and memory impairments mimicking PTSD symptoms in SPS rats. **(A)** Representative travel trajectories of rats in the OFT, **(B)** EPMT, **(C)** three-chamber SIT, and **(D)** MWM. **(E)** Number of entries into the center and **(F)** percentage of total movement spent in the center for the rats in each group during the OFT. **(G)** Percentage of entries into the open arm and **(H)** percentage of time spent in the open arm for the rats in each group in the EPMT. **(I)** Time spent by the tested rats in chamber U1. **(J)** Time spent by the tested rats in chamber U2. **(K)** Time the tested rats spent sniffing U1 rats. **(L)** Time the tested rats spent sniffing U2 rats. **(M)** Escape latency of the rats in each group on different test days. **(N)** Percentage of time the rats stayed in the target quadrant. The study used twenty rats per group. The data are represented as the mean ± SE. The data were analyzed using one-way ANOVAs followed by LSD post hoc tests; * indicates *p* < 0.05, ** indicates *p* < 0.01, and *** indicates *p* < 0.001, compared to the Veh group. # indicates *p* < 0.05, ## indicates *p* < 0.01, and ### indicates *p* < 0.001, the SPS + AR group vs. SPS group.

Rats that underwent SPS exhibited PTSD-like behaviors manifested as reduced activity in the central area of the OFT, reduced activity in the open arm of the EPMT, decreased sociability and preference for social novelties in the SIT, and impaired spatial learning and memory in the MWM test (Figure 2) (Zhao et al., 2020; Alzoubi et al., 2022; Sun et al., 2022). All of these behaviors suggestive of anxiety, avoidance, fear, and memory impairment were used as indicators to validate the establishment of the PTSD animal model. Administration of AR alleviated the manifestations of anxiety, depression, and memory impairment.

### 3.2 Effects of AR administration on SPS-induced anxiety-like behaviors

OFT is often used to assess anxiety in laboratory animals (Sadeghi et al., 2024); anxiety in the tested animals is reflected by their total entries into the field and the amount of time they spend in the center of the arena (Zhao et al., 2022), where higher degrees of anxiety are associated with less open-field exploration and more cautious behaviors (Zhang et al., 2021). The number of entries into the center and percentage of total movement spent in the center for the rats in four groups differed statistically significantly [(F (3, 76) = 3.481, *p* = 0.02) and (F (3, 76) = 4.249, *p* = 0.008), respectively]. The data underwent a detailed analysis using the LSD *post hoc* test. In comparison rats in the Veh group, the SPS rats entered the center region significantly less often (LSD *post hoc* test, *p* = 0.004, Figure 2E) and traveled a significantly shorter distance in the central region during the OFT (LSD *post hoc* test, *p* = 0.04, Figure 2F). These two indicators significantly improved with the administration of AR—i.e., in the SPS + AR group (LSD *post hoc* test, *p* = 0.027 and *p* = 0.002, respectively).

Like the OFT, the EPMT is commonly used to monitor anxiety in laboratory animals. Specifically, the amount of time the rats spend in the two closed arms of the apparatus *versus* its two open arms, as well as the frequency of entries into the latter, can be used to assess their degree of anxiety (Qu et al., 2021; Sun et al., 2022). Higher degrees of anxiety are most often associated with less time spent in the open arms and more time spent in the closed arms (Berardi et al., 2014). Both the percentage of entries into the open arm and the percentage of time spent in it are statistically significant [(F (3, 76) = 3.481, *p* = 0.02, Figure 2G); (F (3, 76) = 13.504, *p* < 0.001, Figure 2H); respectively]. Detailed analysis is conducted on each group using the LSD *post hoc* test. The SPS rats made fewer entries into the open arms and spent notably less time in the open arms relative to the Veh group (LSD *post hoc* test, *p* < 0.001 and *p* < 0.001, respectively). However, AR administration in the SPS + AR group dampened this trend when compared to the SPS group for both indicators mentioned above (*post hoc* test, SPS + AR vs. SPS, *p* = 0.003 and *p* = 0.049, respectively).

### 3.3 Effects of AR administration on social interaction behaviors

The social behaviors and sociability of the rat models of PTSD were evaluated using the three-chamber SIT (Zhao et al., 2020; Zhao et al., 2022): a common assessment of the severity of PTSD-like symptoms such as social withdrawal and avoidance (Zhao et al., 2020). This test is also used to evaluate the efficacy of potential PTSD treatments by measuring changes in social behaviors and sociability following intervention (Mizrachi et al., 2022; Zhao et al., 2022). As shown in Figures 2C, I–L, significant differences are observed in all four indices of the three-chamber SIT across all four groups [Time spent by the tested rats in chamber U1: (F (3, 76) = 6.413, *p* < 0.001); Time spent by the tested rats in chamber U2: (F (3, 76) = 5.929, *p* = 0.001); Time the tested rats spent sniffing U1 rats: (F (3, 76) = 4.491, *p* = 0.006); Time the tested rats spent sniffing U2 rats: (F (3, 76) = 5.774, *p* = 0.001)]. Following this, detailed analyses are conducted using LSD *post hoc* tests. SPS rats spent significantly less time in chambers U1 or U2 relative to rats in the Veh group (*p* = 0.002 and *p* < 0.001, respectively). The time spent in chambers U1 and U2 increased significantly following the administration of AR (SPS + AR vs. SPS, *p* = 0.04 and *p* = 0.027, respectively). Consistently, as shown in Figures 2C, K, L, SPS rats spent significantly less time sniffing in chambers U1 or U2 than rats in the Veh group (LSD *post hoc* test, *p* < 0.001 and *p* = 0.007, respectively). AR administration significantly increased the sniffing time spent in chambers U1 and U2 (LSD *post hoc* test, in the SPS + AR vs. SPS, *p* = 0.045 and *p* = 0.05, respectively).

### 3.4 Effects of AR administration on learning and memory behaviors

The MWM test can help to detect deficits in learning, memory, and spatial navigation in murine models of PTSD and evaluate the efficacy of potential PTSD treatments (Wen et al., 2016; Liu et al., 2020; Niu et al., 2022). Specifically, the MWM test assesses cognitive impairment based on the animal’s ability to navigate to a submerged platform using memory and spatial cues (Mizrachi et al., 2022). Hence, if a drug or therapy alleviates the spatial learning and memory deficits in animals with PTSD-like symptoms as demonstrated by the MWM test, the intervention shows promise as a treatment for humans with PTSD (Mizrachi et al., 2022). The results presented in Figures 2D, M, N indicate that AR administration achieved a significant improvement in SPS-induced cognitive impairment. With grouping (Veh, AR, SPS + AR, SPS) as the between-subjects factor and test point (D1-D5) as the within-subjects factor, a two-way repeated measures ANOVA determined a significant effect of both grouping and test sessions (D1-D5) on the performance in the MWM [F (3, 76) = 28.909, *p* < 0.001 for between-subjects effects, F (4, 76) = 1654.42, *p* < 0.001 for within-subjects effects]. An interaction between grouping and test session was also observed [F (4, 76) = 20.65, *p* < 0.001]. Furthermore, when employing the LSD *post hoc* test, it was observed that the escape latencies were significantly prolonged in the SPS rats in comparison to the Veh rats (*p* < 0.001). As anticipated, the administration of AR for the SPS + AR rats mitigated this behavior in comparison to the SPS rats (*p* < 0.001). Afterwards, the percentage of time the rats stayed in the target quadrant differed significantly among the four groups (F (3, 76) = 11.416, *p* < 0.001, Figure 2N). Subsequently, the SPS group spent significantly less time exploring the quadrant containing the target platform than did the Veh group (LSD *post hoc* test, *p* < 0.001). Importantly, AR administration significantly increased the amount of time spent staying in the target quadrant (LSD *post hoc* test, SPS + AR vs. SPS, *p* = 0.045).

### 3.5 Effects of AR administration on the expression of synaptic plasticity-related proteins

**FIGURE 3:**
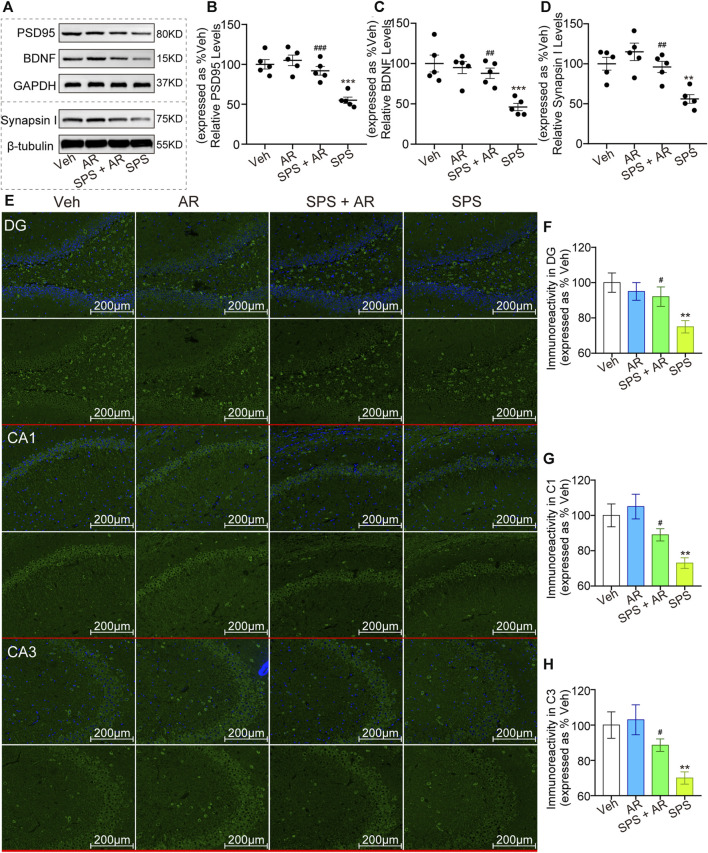
AR administration increased the levels of synaptic plasticity-related proteins PSD95, BDNF, and Synapsin I in rats that underwent SPS. **(A)** Representative immunoblots of hippocampal BDNF, PSD95, and Synapsin I for the four groups of rats. **(B–D)** Semi-quantitative analysis of expressions of PSD95, BDNF, and Synapsin I. **(E)** Representative immunofluorescence images of BDNF in the DG, C1, and C3 regions of the hippocampus. **(F–H)** Immunoreactivity analysis of BDNF in DG, CA1, CA3, respectively. Scale bars: 200 µm. The study used five rats per group. The data are represented as the mean ± SE. The data were analyzed using one-way ANOVA followed by an LSD *post hoc* test; * indicates *p* < 0.05, ** indicates *p* < 0.01, and *** indicates *p* < 0.001, compared to the Veh group. # indicates *p* < 0.05, ## indicates *p* < 0.01, and ### indicates *p* < 0.001, the SPS + AR group vs. SPS group.

As shown in Figures 3A–D, Western blotting was performed to evaluate the levels of synaptic plasticity-associated proteins PSD95, BDNF, and Synapsin I in each group of rats. The analysis revealed significant variations in the levels of these proteins between the groups (*p* < 0.001, one-way ANOVA). LSD *post hoc* pairwise comparisons showed that the SPS group exhibited notably lower levels of these proteins when compared to the other groups. Importantly, relative to the SPS group, the SPS + AR group exhibited a significant elevation in these protein levels (PSD95: *p* = 0.005; BDNF: *p* = 0.006; Synapsin I: *p* = 0.014; LSD *post hoc*). Consistently, as shown in Figures 3E–H, the results of the immunofluorescence assay showed significant differences in the fluorescence intensity of BDNF in the four groups (DG: F (3, 16) = 4.819, *p* = 0.014; CA1: F (3, 16) = 7.144, *p* = 0.003; CA3: F (3, 16) = 5.842, *p* = 0.007). The fluorescence intensity of BDNF protein was significantly lower in SPS rats compared to rats in the Veh group, as indicated by the LSD *post hoc* test (DG: *p* = 0.003; CA1: *p* = 0.002; CA3: *p* = 0.003). Administration of AR significantly reversed this trend, with *p* values of 0.026, 0.049, and 0.049 for DG, CA1, and CA3, respectively, in the SPS + AR group compared to the SPS group (LSD *post hoc* test).

### 3.6 Effects of AR admin istration on the ultrastructure of synapses in the CA1 following SPS

**FIGURE 4:**
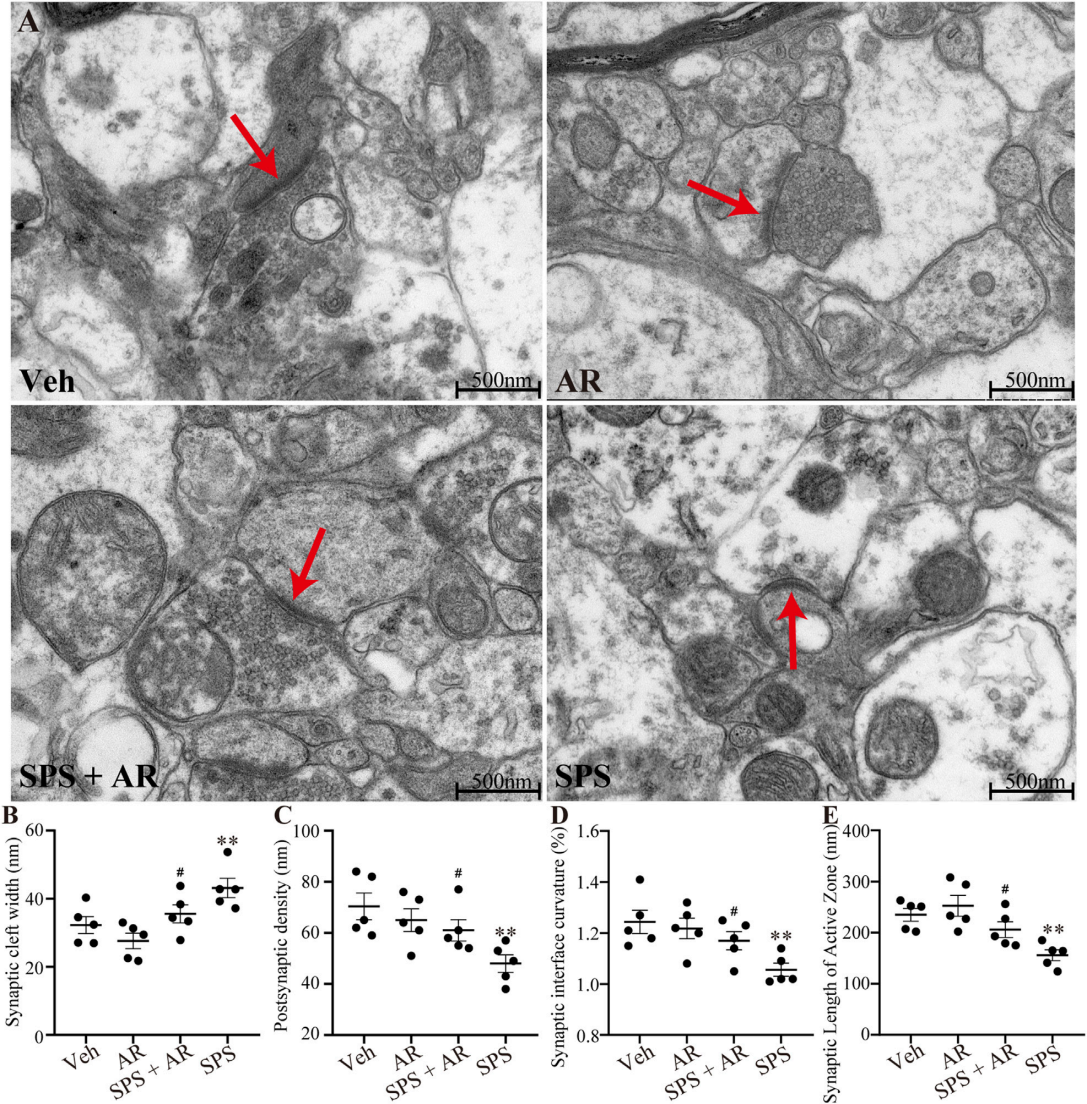
Changes in the ultrastructure of synapses in the CA1 region in five images randomly selected from each group for analysis with TEM. **(A)** Representative images of synapses in the hippocampal CA1 region for the four groups of rats. **(B–E)** The synaptic cleft width, postsynaptic density, synaptic interface curvature, and synaptic length of the active zone in CA1. The study used five rats per group. Red arrows indicate presynaptic membranes. The data are represented as the mean ± SE. The data were analyzed using one-way ANOVAs followed by LSD *post hoc* tests; * indicates *p* < 0.05, ** indicates *p* < 0.01, and *** indicates *p* < 0.001, compared to the Veh group. # indicates *p* < 0.05, the SPS + AR group vs. SPS group.

As shown in Figures 4A–E, ultrastructural observations observed under TEM revealed significant differences in the synaptic cleft width, postsynaptic density, synaptic interface curvature, and synaptic length of the active zone in CA1 between the different groups of rats (F (3, 16) = 6.408, *p* = 0.005; F (3, 16) = 4.766, *p* = 0.014; F (3, 16) = 4.894, *p* = 0.013; F (3, 16) = 7.716, *p* = 0.002, respectively, one-way ANOVA). Further analysis with LSD *post hoc* tests identified significant differences in the aforementioned four indicators between the SPS and the SPS + AR groups (synaptic cleft width, *p* = 0.049; postsynaptic density, *p* = 0.045; synaptic interface curvature, *p* = 0.048; and synaptic length of the active zone, *p* = 0.033).

### 3.7 Effect of AR administration on apoptosis of HIP neurons following SPS

**FIGURE 5:**
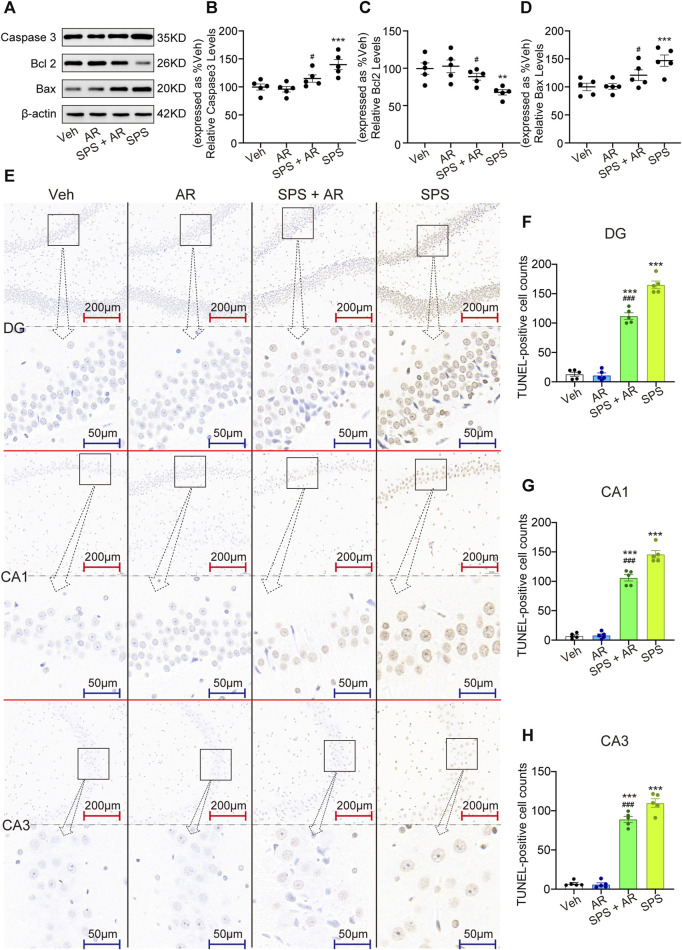
AR administration attenuates neuroapoptosis in rats that underwent SPS. **(A)** Representative immunoblots of hippocampal Caspase 3, Bcl 2, and Bax for the four groups of rats. **(B–D)** Semi-quantitative analysis of Caspase 3, Bcl 2, and Bax expression. **(E)** Representative images of TUNEL staining of the hippocampal DG, C1, and C3 regions for each group. **(F–H)** Changes in apoptotic cells for each group as revealed by TUNEL staining of the hippocampus. The study used five rats per group. The data are represented as the mean ± SE. The data were analyzed using one-way ANOVAs followed by an LSD *post hoc* test; * indicates *p* < 0.05, ** indicates *p* < 0.01, and *** indicates *p* < 0.001, compared to the Veh group. # indicates *p* < 0.05, ## indicates *p* < 0.01, and ### indicates *p* < 0.001, the SPS + AR group vs. SPS group.

As shown in Figures 5A–D, Western blotting was used to evaluate the expression of apoptosis regulators Caspase 3, Bcl 2, and Bax in the hippocampal neurons of rats in each of the four groups. The analysis revealed significant variations in protein levels between the groups (Caspase 3, F (3, 16) = 8.894, *p* = 0.001; Bcl 2, F (3, 16) = 6.244, *p* = 0.005; and Bax, F (3, 16) = 7.563, *p* = 0.002; one-way ANOVA). LSD *post hoc* pairwise comparisons showed that the SPS group exhibited notably lower levels of these proteins when compared to the Veh group (Caspase 3, *p* = 0.001; Bcl 2, *p* = 0.005, Bax, *p* = 0.002). Administration of AR significantly reversed this trend, with *p* values of 0.018, 0.033, and 0.036 for Caspase 3, Bcl 2, Bax, respectively, in the SPS + AR group compared to the SPS group (LSD *post hoc* test). As shown in Figures 5E–H, the results TUNEL staining experiment agreed with those obtained from the Western blots: significant differences were found in the number of TUNEL-positive cells in the three subregions of the hippocampus between different groups (DG: F (3, 16) = 230.36, *p* < 0.001; CA1: F (3, 16) = 239.253, *p* < 0.001; CA3: F (3, 16) = 225.306, *p* < 0.001). The fluorescence intensity of BDNF protein was significantly lower in SPS rats compared to rats in the Veh group, as indicated by the LSD *post hoc* test (DG: *p* = 0.003; CA1: *p* = 0.002; CA3: *p* = 0.003). LSD *post hoc* tests showed that the number of TUNEL-positive cells was significantly increased in three subregions of the hippocampus of rats in the SPS group compared with rats in the Veh group (three hippocampal subregions: *p* < 0.001). Administration of AR significantly reversed this trend, with *p*-values less than 0.001 for DG, CA1, and CA3 in the SPS + AR group compared to the SPS group.

**FIGURE 6:**
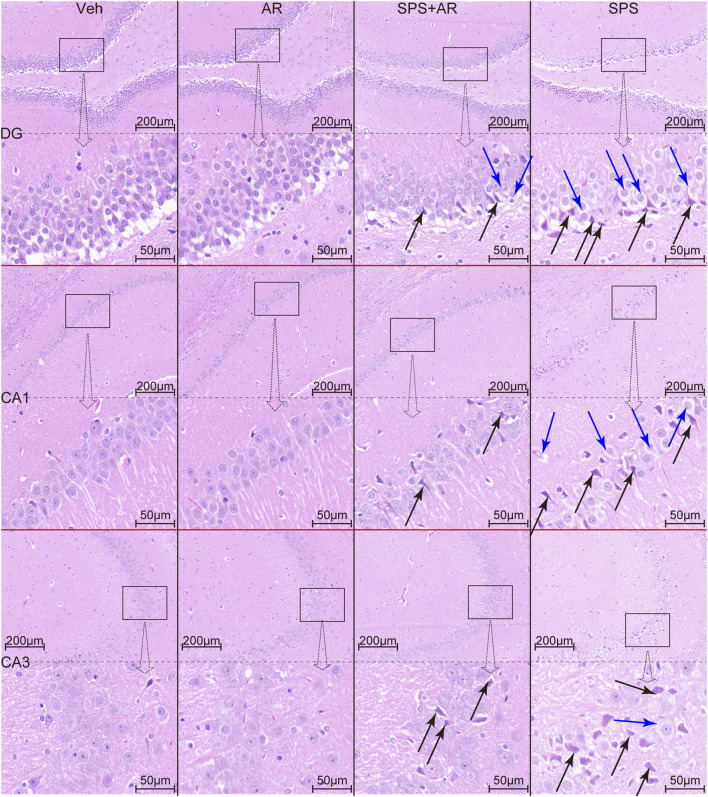
HE staining reveals the histopathological changes in hippocampal neurons of rats in each group. Black arrows indicate neuronal consolidation, deep staining, and increased basophilia. Blue arrows indicate neuronal hydropic degeneration and cytoplasmic vacuolization.

Finally, HE staining was performed to identify any pathological changes in the hippocampal brain tissue samples obtained from each group. We observed shrinkage, deep staining, unclear demarcation between the nucleus and cytoplasm, enhanced basophilia, and sparse cell arrangement (indicated by black arrows in Figure 6) in neurons in the hippocampal DG region, CA1 region, and CA3 region of rats in the SPS group. We also found significant neuronal hydropic degeneration and cytoplasmic vacuolization (indicated by blue arrows in Figure 6). AR administration significantly attenuated the histopathological damage and improved vacuolization.

## 4 Discussion

The present study evaluated the effects of AR on PTSD-like behavioral phenotypes, hippocampal synaptic plasticity, and hippocampal neuronal apoptosis in rats subjected to SPS. The administration of AR to SPS rats is herein demonstrated to enhance OFT central area activity and EPMT open arm activity, increase sociability and preference for social novelty in the SIT, and alleviate impaired spatial learning and memory in the MWM test. This evidence suggests that AR may improve symptoms related to PTSD—particularly anxiety, social functional impairment, and cognitive dysfunction. The present study further observed that administering AR to SPS rats improved synaptic plasticity by upregulating synaptic plasticity-related proteins PSD95, BDNF, and Synapsin I. The reduction of SPS-induced neuronal apoptosis is consistent with AR’s known anti-apoptotic properties. A growing body of evidence demonstrates that AR is a multifunctional neuroprotective agent that may be used to treat PTSD.

The present study observed significant anxiety-like behavior, social interaction deficits, and impaired learning and memory in rats subjected to SPS. Consistent with the hypothesis of this study, AR administration to rats that underwent SPS was found to attenuate the behavioral consequences of SPS. While the “SPS + intervention” behavioral pharmacology paradigm adopted in this investigation has featured broad use in the literature in many studies, our use of AR as the intervention is relatively unique: e.g., Chen YL et al. (PMID: 35259352)found that MiR-153 downregulation alleviates PTSD-like behaviors in SPS rats (Chen et al., 2022), while Chen Y et al. (PMID: 35753367) observed that MicroRNA-124 attenuates PTSD-like behaviors in SPS rats (Chen et al., 2022). These reports fully demonstrate that the behavioral pharmacology paradigm of “SPS + intervention” is well-established in the study of the pathogenesis and possible treatment of PTSD. Hence, the successful construction of a rat model of simulated PTSD with SPS in the present study helps to validate our findings concerning the therapeutic benefits of AR.

While maladaptive social interactions are common among patients with PTSD (Collimore et al., 2010; Stevens and Jovanovic, 2019; Bjornsson et al., 2020; Sharma et al., 2020; Laban et al., 2021), most research on the behavioral characterization of SPS rats has focused on anxiety-like behaviors and learning memory deficits (Schoner et al., 2017; Hou et al., 2021; Lo et al., 2023). Hence, the present investigation was further innovative in its focus on impaired social interactions in SPS rats. We found that SPS intervention induced social impairment and social novelty preference disorder. AR administration attenuated the social impairment and social novelty preference disorder induced by SPS intervention.

PTSD usually results in social impairment due to several interrelated factors. First, patients with PTSD experience strong anxiety and fear responses in social situations (Collimore et al., 2010; Bjornsson et al., 2020). Our observation that SPS rats avoided social interactions and spent less time in the central area of the OFT and the open arm in of the EPMT reflects the above-mentioned symptoms in patients with PTSD. Second, the cognitive deficits associated with PTSD—which leads to diminished attention and focus that exacerbates difficulties in social interactions—are paralleled by the impaired spatial navigational learning memory demonstrated by the SPS rats in the MWM (Stevens and Jovanovic, 2019). The methodology of the present study underscores the need to standardize investigations of animal sociability in basic research on PTSD. Furthermore, as demonstrated by our findings, studying animal sociability helps to assess the effect of interventions (in this study, AR administration) on the integrated treatment of PTSD.

The present investigation observed that administering AR to rats that underwent SPS significantly increases the expression of synaptic plasticity-related proteins PSD95, BDNF, and Synapsin I in the hippocampus, positively affects synaptic ultrastructure, and inhibits neuronal apoptosis. In the context of our behavioral experiments that confirm that AR significantly alleviates PTSD-like phenotype in SPS rats, these molecular studies suggest that AR’s neuroprotective effects in SPS rats may be ascribed to its positive effects on synaptic plasticity and inhibition of neuronal apoptosis. A large number of preclinical studies have investigated the therapeutic effects of drugs or other therapeutic strategies on PTSD symptoms by studying changes in synaptic plasticity and neuronal apoptosis (Li et al., 2013; Ji et al., 2019; Feng et al., 2020; Jiang et al., 2022; Li et al., 2022): e.g., Sevoflurane has been shown to attenuate apoptosis and improve synaptic plasticity in the hippocampus of rat models of PTSD (Gu et al., 2023), and down-regulating MiR-153 has been observed to alleviate PTSD-like behaviors by affecting synaptic plasticity and apoptosis (Chen et al., 2022). Hence, the present findings that AR enhances synaptic plasticity and inhibits neuronal apoptosis suggest that it has potential as an effective therapeutic agent in the treatment of PTSD.

Although this study is, to the best of our knowledge, the first to investigate whether AR has a therapeutic effect on PTSD-like symptoms, a large body of literature has reported that AR improves synaptic plasticity and attenuates neuronal apoptosis in models of multiple neurodegenerative disorders, including Parkinson’s disease, Alzheimer’s disease, Huntington’s disease, and multiple sclerosis (Zhao et al., 2020; Arthur et al., 2023). Poorgholam et al. found that artemisinin improved synaptic plasticity in rat models of Alzheimer’s disease and diabetes (Poorgholam et al., 2023), while Xia et al. observed that administering Dihydroartemisinin could have a therapeutic effect on impaired synaptic plasticity caused by tauopathies (Xia et al., 2021). This study expands upon this understanding of the use of AR in promoting neuroprotection by expanding its potential clinical application to the treatment of PTSD.

The present study is subject to two limitations. First, we only considered the neurobiological changes in the rat hippocampus. While this was justified by our focus on investigating the mechanism by which AR affects the behavior of SPS rats, other brain regions (e.g., prefrontal cortex and amygdala) are also involved in the acquisition of fear memories (Shin et al., 2006; Alexandra et al., 2022; Ressler et al., 2022). Research on SPS-induced molecular and biological changes in other neural regions, as well as the effect of AR on these changes in those regions, should also garner our necessary attention. Second, we prioritized the study of AR’s effects on synaptic plasticity and neuronal apoptosis without sufficiently investigating other factors contributing to the pathogenesis of PTSD, such as neuroinflammation or epigenetic changes (Al et al., 2021; Lv et al., 2023). A comprehensive understanding of the effects of AR on these factors could bolster support for its potential as a therapeutic agent.

## 5 Conclusion

By confirming that AR improves synaptic plasticity and inhibits neuronal apoptosis in a rat model of PTSD, the present report provides evidence for the therapeutic potential of AR in the treatment of PTSD. Our findings further contribute to a greater understanding of the multifaceted neuroprotective effects of AR and provide the necessary preclinical data to support future investigation of its application to the treatment of PTSD.

## Data Availability

The original contributions presented in the study are included in the article/Supplementary material, further inquiries can be directed to the corresponding authors.
