# Corrigendum: Artemisinin reduces PTSD-like symptoms, improves synaptic plasticity, and inhibits apoptosis in rats subjected to single prolonged stress

**DOI:** 10.3389/fphar.2024.1427681

**Published:** 2024-06-18

**Authors:** Qing Liu, Xiaoyan Ding, Ying Wang, Hairong Chu, Yan Guan, Meng Li, Kuisheng Sun

**Affiliations:** School of Laboratory Medicine, Weifang Medical University, Weifang, Shandong, China

**Keywords:** post-traumatic stress disorder, artemisinin, single prolonged stress, synaptic plasticity, apoptosis

In the published article, there was an error in (Figure 2) as published.

**FIGURE 2:**
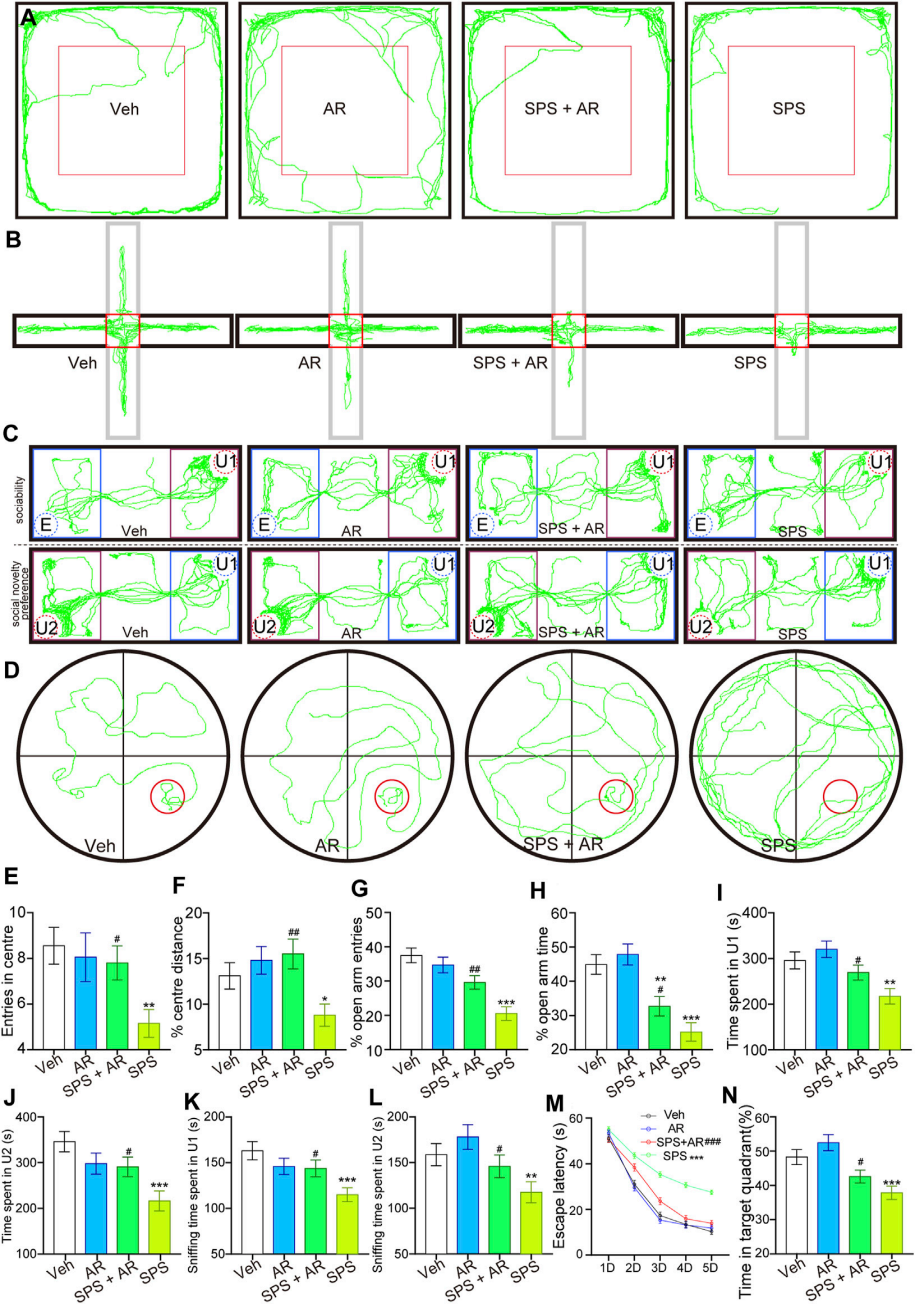
AR administration mitigated anxiety-like behaviors, social aversion, and learning and memory impairments mimicking PTSD symptoms in SPS rats. **(A)** Representative travel trajectories of rats in the OFT, **(B)** EPMT, **(C)** three-chamber SIT, and **(D)** MWM. **(E)** Number of entries into the center and **(F)** percentage of total movement spent in the center for the rats in each group during the OFT. **(G)** Percentage of entries into the open arm and **(H)** percentage of time spent in the open arm for the rats in each group in the EPMT. **(I)** Time spent by the tested rats in chamber U1. **(J)** Time spent by the tested rats in chamber U2. **(K)** Time the tested rats spent sniffing U1 rats. **(L)** Time the tested rats spent sniffing U2 rats. **(M)** Escape latency of the rats in each group on different test days. **(N)** Percentage of time the rats stayed in the target quadrant. The study used twenty rats per group. The data are represented as the mean ± SE. The data were analyzed using one-way ANOVAs followed by LSD post hoc tests; * indicates *p* < 0.05, ** indicates *p* < 0.01, and *** indicates *p* < 0.001, compared to the Veh group. # indicates p < 0.05, ## indicates *p* < 0.01, and ### indicates *p* < 0.001, the SPS + AR group vs. SPS group.

In the original Figure 2M, the line graph incorrectly displayed mean ± SD; however, the accurate line graph should depict mean ± SE. The corrected graphs now exhibit data in the format of “Mean ± SE.”

The corrected Figure 2 and its caption appear below.

The authors apologize for this error and state that this does not change the scientific conclusions of the article in any way. The original article has been updated.

